# Conjunctival allergic inflammation involving the interleukin-23/T helper type 17 immune axis in an experimental allergic conjunctivitis mouse model

**DOI:** 10.1007/s10384-026-01333-4

**Published:** 2026-02-13

**Authors:** Rumi Adachi, Jun Shoji, Noriko Inada, Akiko Tomioka, Satoru Yamagami

**Affiliations:** Division of Ophthalmology, Department of Visual Sciences, Nihon University School of Medicine, 30-1 Oyaguchi-Kamichou, Itabashi-ku, Tokyo, 173-8610 Japan

**Keywords:** Allergic conjunctivitis, Interleukin-23, Experimental mouse model, Eosinophil infiltration, T helper 17 cells

## Abstract

**Purpose:**

To investigate the role of the interleukin-23 (IL-23)/T helper type 17 (Th17) immune axis in conjunctival allergic inflammation using a murine model of experimental allergic conjunctivitis (EAC).

**Study design:**

Experimental study.

**Methods:**

BALB/c mice were assigned to four groups: control (untreated), allergy (EAC), IL-23 (EAC with IL-23 eyelid injection), and non-sensitized IL-23 (non-sensitized with IL-23 eyelid injection). Conjunctival tissue was analyzed histologically to quantify eosinophil and neutrophil infiltration. Gene expression of mRNA in conjunctival tissue was assessed using a PCR array and quantitative RT-PCR.

**Results:**

Eosinophilic and neutrophilic infiltration in the subconjunctival tissue was more pronounced in the IL-23 group than in the allergy and control groups. PCR array and quantitative RT-PCR analyses revealed significantly elevated *Ccl17/Tarc* mRNA expression in the IL-23 group compared to the allergy group. *IL-17A* mRNA, undetectable in the allergy group, was expressed in the IL-23 group. Additionally, PCR array comparisons between the IL-23 and non-sensitized IL-23 groups showed a significant increase in *Rorc* and *Il1r1* expression in the IL-23 group. At the same time, *Mmp3*, *Ccl7*, and *Ccr2* were significantly upregulated in the non-sensitized IL-23 group. RT-PCR analysis also demonstrated higher *IL-17A* mRNA levels in the non-sensitized IL-23 group than in the IL-23 group.

**Conclusion:**

IL-23 induces mixed eosinophilic–neutrophilic inflammation in conjunctival tissue, characterized by enhanced Th2 and weak Th17 responses in a murine model of EAC. These findings suggest a modulatory role of the IL-23/Th17 axis in influencing the severity and phenotype of allergic conjunctival inflammation.

## Introduction

Allergic conjunctival diseases (ACDs) are inflammatory disorders of the conjunctiva primarily characterized by immediate hypersensitivity reactions [1, 2]. ACDs include allergic conjunctivitis (AC), atopic keratoconjunctivitis (AKC), and vernal keratoconjunctivitis (VKC), each with distinct clinical features and varying degrees of severity [2]. Type 2 inflammation mediated by T helper type 2 (Th2) cells and group 2 innate lymphoid cells (ILC2) is a shared pathogenic mechanism in ACDs [3, 4]; however, the immunological mechanisms underlying their diverse clinical presentations and severity remain poorly understood.

In addition to the eosinophilic infiltration induced by type 2 inflammation in target organs, enhanced neutrophilic infiltration is reported in severe allergic conditions. In patients with asthma, disease severity correlates with increased airway neutrophilia and elevated levels of neutrophil-attracting chemokines such as interleukin (IL)-8 and IL-17 [5]. Our previous work demonstrates that analysis of chemokine mRNA expression in the ocular surface in chronic AC, including AKC and VKC, showed increased expression of both IL-8 (associated with neutrophil infiltration) and eotaxin-2 (associated with eosinophil infiltration) [6]. Th2 responses are dominant in severe allergic inflammation; however, accumulating evidence indicates that Th17-mediated immune responses may also contribute to disease severity. In a murine model of airway inflammation, IL-23 and Th17 cells not only drove neutrophilic inflammation but also upregulated Th2-mediated eosinophilic responses [7]. Similarly, in a murine model of experimental AC (EAC), an exaggerated Th17 response aggravated Th2-dominant allergic inflammation in the conjunctiva of IL-27Rα-deficient mice [8]. These findings support the hypothesis that mixed eosinophilic and neutrophilic inflammation, along with a complex interplay among Th1, Th2, and Th17 cytokine profiles in conjunctival tissues, may modulate the immunopathogenesis and contribute to the severity and refractoriness of ACDs.

IL-23, a member of the IL-12 cytokine family, is composed of the IL-23p19 and IL-12p40 subunits [9]. IL-23 plays a multifaceted role in immunity, including host defense against bacterial and fungal pathogens, maintenance of homeostasis, induction of autoimmune reactions by IL-23 overexpression, and inflammatory responses via the IL-23/Th17 axis [10–12]. It is a proinflammatory cytokine predominantly secreted by dendritic cells and activated macrophages [13], and serves as a critical regulator in both innate and adaptive immune responses [14]. IL-23 also induces the differentiation, expansion, and effector function of Th17 cells. The development of pathogenic Th17 cells is influenced by the surrounding cytokine environment, particularly by Th1 (interferon-γ) and Th2 (IL-4) cytokines [15]. Previous studies have examined the Th1/Th2/Th17 balance in autoimmune diseases [16]. However, the contribution of Th17-associated responses to Th2-dominant ACDs remains unclear.

We investigated the histopathological alterations in conjunctival type 2 inflammation in ovalbumin (OVA)-sensitized murine model of AC following local IL-23 injection into the eyelid.

## Materials and methods

### Experimental allergic conjunctivitis mouse model with interleukin-23 eyelid injection

#### Animals and ethical approval

Five-week-old female BALB/c mice were purchased from Charles River, Japan and housed under specific pathogen-free conditions. The mice had unrestricted access to standard food and tap water. All animal experiments were approved by the Animal Care and Use Committee of Nihon University School of Medicine (Approval Number: AP21MED021-1 July,13, 2021) and conducted following the ARVO Statement for the Use of Animals in Ophthalmic and Vision Research.

#### Murine model of experimental allergic conjunctivitis

Mice were randomly divided into four groups (n = 7 per group): control group (untreated mice), allergy group (mice sensitized to induce EAC, followed by eyelid injection of physiological saline), IL-23 group (EAC-induced mice that received eyelid injections of IL-23 solution [1 µg/mL; GTX85738, GeneTex]), and non-sensitized IL-23 group (non-sensitized mice given topical OVA instillation (5 mg/mL) and eyelid IL-23 injections (1 µg/mL).

**Fig. 1:**
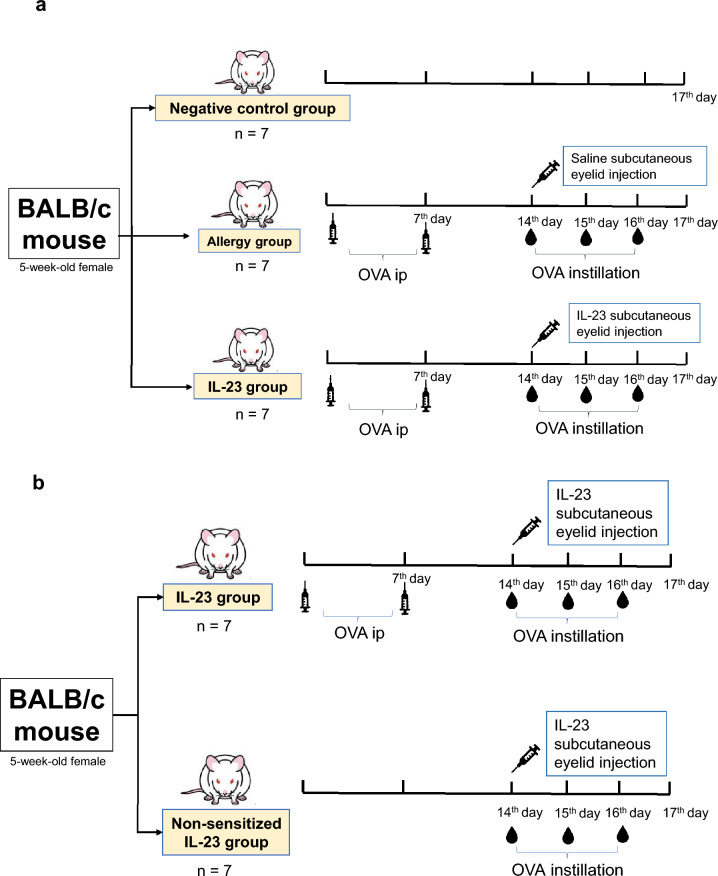
Experimental protocol. **a** Protocol 1 shows the sensitization, eye drop instillation, and eyelid injection procedures for the allergy, interleukin (IL)-23, and control groups. **b** Protocol 2 shows the sensitization, eye drop instillation, and eyelid injection procedures for the IL-23 and non-sensitized IL-23 groups

Figure 1 summarizes two experimental protocols. Protocol 1 (Fig. 1a) assessed the effect of IL-23 on allergic ocular inflammation. Mice in the allergy and IL-23 groups were sensitized with intraperitoneal (i.p.) injection of an emulsion solution of Alum (15 mg/mL; Cosmo Bio Co. Ltd.) and OVA (5 mg/mL; Sigma-Aldrich Japan) twice on days 1 and 7. On day 14, eyelid injections of either physiological (allergy group) or IL-23 solution (1 µg/ml; IL-23 group) were administered subcutaneously into both the upper and lower eyelids. From days 14–16, OVA eye drops were instilled in the mice in the allergy and IL-23 groups in both eyes once daily. The mice were euthanized on day 17, 24 h after the final OVA instillation, by an overdose of pentobarbital. Blood, eyeballs, and eyelids were collected for further analysis.

Protocol 2 (Fig. 1b) investigated the IL-23 response in the presence or absence of allergic inflammation. The mice in the non-sensitized group received eyelid injections of IL-23 on day 14 and topical OVA instillation OU on days 14–16 without i.p. sensitization. The mice were euthanized with pentobarbital on days 17 and 24 h after the final OVA instillation, and blood, eyeballs, and eyelids were collected for further analysis.

### Quantitation of ovalbumin-specific immunoglobulin E

Serum was prepared from whole-blood samples from each mouse. Levels of OVA-specific immunoglobulin E (IgE) antibodies in the serum were quantified using an Anti-Ovalbumin IgE (mouse) EIA Kit (Cayman Chemical), following the manufacturer’s instructions. Samples were diluted (1:50) in phosphate-buffered saline containing 1% bovine serum albumin and 0.05% Tween-20, and quantified within the 1.56-100 ng/ml (assay range)

### Histological and immunohistochemical analysis of conjunctival tissue

The entire conjunctival and corneal tissues from all groups were fixed in 4% paraformaldehyde (WAKO), paraffin-embedded, and sectioned at approximately 6 μm using a microtome (Leica CM1520; Leica Biosystems). Sections were stained with direct fast scarlet (DFS) for eosinophils and processed by immunohistochemistry with the enzyme-labeled antibody method using rat anti-mouse Ly-G6/Ly-C6 (Gr-1) antibodies (BD Pharmingen) with N-Histofine^®^ Simple Stain^TM^ Mouse MAX-PO (Rat) kit (NICHIREI BIOSCIENCES) to identify neutrophils.

#### Eosinophil and neutrophil infiltration in conjunctival tissue

Gr-1- and DFS-stained sections in each group were photographed under a light microscope (BZ-X710; KEYENCE). Subconjunctival tissue areas were quantified in each digital image using a semi-automated image analysis protocol in Adobe Photoshop Elements 14 software (Adobe Systems Inc.). Eosinophil and neutrophil densities (cells/mm^2^) were calculated for each section, and averages were obtained across 15 thin sections per group.

### Laser microdissection quantitative RT-PCR

#### Tissue preparation and mRNA extraction

Conjunctival tissues were embedded in OCT compound (Sakura Seiki) without prior fixation, sectioned at 20 μm, and mounted on RNAse-free polyethylene naphthalate-coated glass slides (Leica Microsystems). Tissue sections were fixed for 30 s in cold 100% methanol, immersed in distilled water, stained with 0.05% toluidine blue, and air-dried. Laser-assisted microdissection (LMD) was immediately performed using the LMD6000 system (Leica Microsystems), targeting subepithelial inflammatory regions for mRNA extraction.

The LMD-dissected samples were collected in Thermo-Tube caps (Thermo Scientific Japan,) containing 10 μL mineral oil (Life Technologies Japan). Total RNA was extracted using the magLEAD^®^ automated system (Hitachi). cDNA was synthesized using the High-Capacity cDNA Reverse Transcription Kit (Life Technologies Japan), and samples were stored at −80°C for further analysis.

#### PCR array and quantitative RT-PCR analysis of conjunctival tissues

For PCR array analysis, conjunctival samples from the allergy and IL-23 groups were used to analyze the expression of 84 genes encoding allergic inflammation-associated factors using the RT2 Profiler™ PCR Array Mouse Allergy & Asthma kit (PAMM-067ZA, Qiagen). Additionally, IL-23 (n = 3) and non-sensitized IL-23 (n = 3) group samples were used to analyze the expression of 84 genes encoding Th17-associated factors using the RT2 Profiler™ PCR Array Mouse Th17 Response kit (PAMM-073ZR, Cat. no. 330231, Qiagen). All procedures followed the manufacturer’s instructions. Fold changes were calculated using the ΔΔCt method with RT2 Profiler™ software (Qiagen).

Quantitative RT-PCR was performed on samples from the allergy, IL-23, and non-sensitized IL-23 groups to evaluate expression levels of IL-5, IL-17A, chemokine (C-X-C motif) ligand (CXCL) 1, and CXCL2. Reactions were performed using TaqMan™ Gene Expression Master Mix (Thermo Fisher Scientific Japan) and predesigned primers/probes, including IL-5 (*il5*, Mm00439646_ml), IL-17A (*il17a*, Mm00439618_m1), CXCL1 (*cxcl1*, Mm04207460_m1), and CXCL2 (*cxcl2*, Mm00436450_ml) (Life Technologies Japan), on a StepOnePlus^TM^ system (Life Technologies Japan). Gene expression was normalized to β-*actin* (Mm000607939_s1), and relative expression was calculated using the ∆∆Ct method.

### Statistical analysis

Eosinophil and neutrophil densities across the control, allergy, and IL-23 groups were compared using the nonparametric Steel–Dwass test. Differences in mRNA expression between the allergy and IL-23 groups, and between the IL-23 and non-sensitized IL-23 groups, were analyzed using a parametric *t*-test. Statistical significance was set at *p* < 0.05. All statistical analyses were performed using the MAC Toukei–Kaiseki version 3 software (Esumi).

## Results

### Serum levels of OVA-specific IgE antibodies

**Fig. 2:**
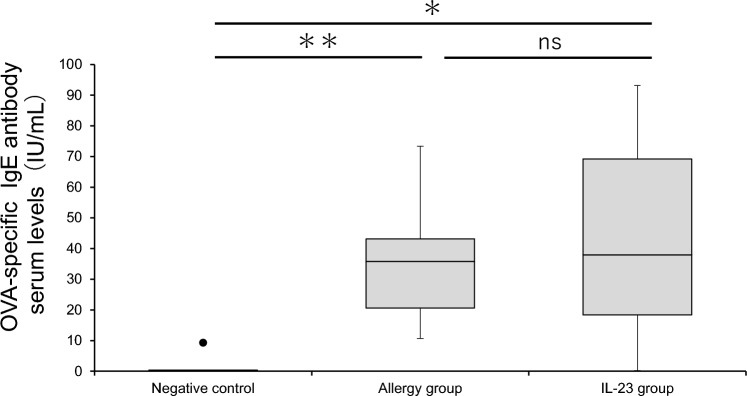
Ovalbumin (OVA)-specific immunoglobulin E (IgE) antibody levels in serum. OVA-specific IgE antibody levels in the allergy and interleukin-23 groups are significantly higher than those in the negative control group. ^*^p < 0.05, ^**^p < 0.01; NS, not significant; Steel–Dwass test

Serum levels of OVA-specific IgE antibodies were significantly higher in both the allergy (p < 0.01) and IL-23 (*p* < 0.05) groups compared with the control group (Fig. 2). These results confirm that OVA sensitization was established in the allergy and IL-23 groups.

### Histological and immunohistochemical findings in conjunctival tissue

#### Neutrophilic infiltration

**Fig. 3:**
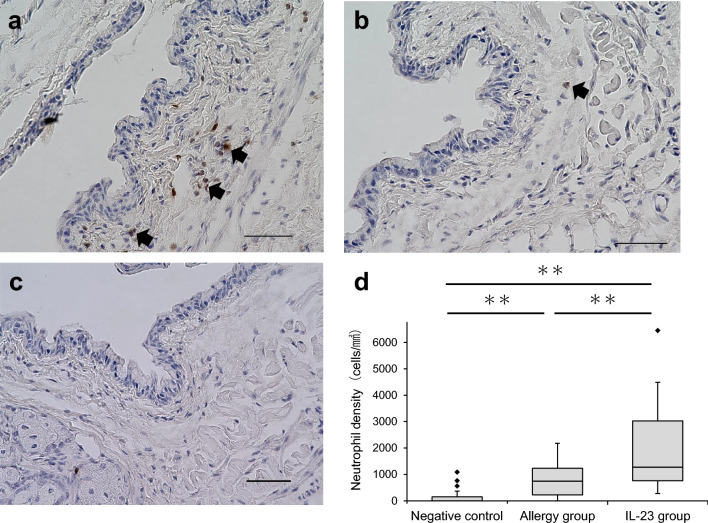
Histological analysis of neutrophils in the conjunctiva. Light microscopy photographs of the conjunctiva in the **a** interleukin (IL)-23 groups, and black arrows indicate neutrophils, **b** allergy, and **c** control, **d** Quantitative analysis of neutrophil density in the subconjunctival tissue in the control, allergy, and IL-23 groups. Neutrophil density in the IL-23 group is significantly higher than that in the allergy and control groups. ^**^ *p* < 0.01, Steel–Dwass test

Based on Gr-1 immunostaining (experimental protocol 1), increased neutrophilic infiltration was more severe in the subconjunctival tissues of the IL-23 group (*p* < 0.01, Fig. 3a) compared with the allergy group (Fig. 3b). It was absent in the control group (Fig. 3c). Quantitative analysis revealed that neutrophil density in both the allergy (*p* < 0.01) and IL-23 (*p* < 0.01) groups was significantly higher than in the control group, with the IL-23 group (*p* < 0.01) exhibiting a substantially greater density than the allergy group (Fig. 3d).

#### Eosinophilic infiltration

**Fig. 4:**
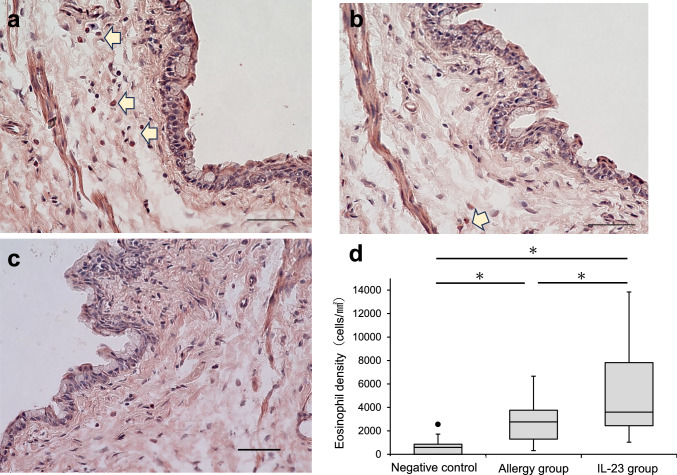
Histological analysis of eosinophils in the conjunctiva. Light microscopy photographs of the conjunctiva in the **a** interleukin (IL)-23 groups, with eosinophils indicated by yellow arrows, **b** allergy, and **c** control, **d** Quantitative analysis of eosinophil density in the subconjunctival tissue in the control, allergy, and IL-23 groups. The eosinophil density in the IL-23 group is significantly higher than that in the allergy and control groups. ^*^p < 0.05, Steel–Dwass test

DFS staining showed marked eosinophil infiltration in subconjunctival tissues in the IL-23 and allergy groups (Fig. 4a and b), while eosinophils were absent in the control group (Fig. 4c). Quantitative analysis demonstrated significantly higher eosinophil densities in the allergy (*p* < 0.05) and IL-23 (*p* < 0.05) groups relative to the control group, and eosinophil density was significantly higher in the IL-23 group (*p* < 0.05) than in the allergy group (Fig. 4d).

### PCR array and quantitative PCR analyses of conjunctival tissue

#### PCR array

To investigate gene expression profiles associated with allergic inflammation (experimental protocol 1). The IL-23 group demonstrated a significantly higher fold increase in thymus and activation-regulated chemokine (Ccl17/Tarc) expression compared with the allergy group (Table 1). The PCR array results for eosinophil-associated chemokines (Table 2). No difference in the expression levels of CCL11/eotaxin-1, CCL24/eotaxin-2, and CCL26/eotaxin-3 mRNA was observed between the allergy group and the IL-23 group.

**Table 1:** Comparison of type 2 inflammation-related factors in the interleukin (IL)-23 group versus the allergy group

Symbol	Fold increase (IL-23 vs. Allergy group)	*p* value
*Il1rl1/ST2*	6.60	0.328
*Mmp9*	5.78	0.166
*Ccl12/MCP-5*	5.08	0.167
*Ccl17/TARC*	3.10	0.032*
*Ccl22/MDC*	1.81	0.360

^*^ *p* <0.05

*Il1rl1* interleukin 1 receptor-like 1; *Mmp9* matrix metalloproteinase-9; *Ccl2* C-C motif chemokine ligand 12; *MCP-5* monocyte chemotactic protein 5; *Ccl17* C-C motif chemokine 17; *TARC* thymus and activation-regulated chemokine; *Ccl22* C-C motif chemokine 22; *MDC* macrophage-derived chemokine

**Table 2:** Comparison of eosinophil-associated factors in the interleukin (IL)-23 group versus the allergy group

Symbol	Fold increase (IL-23 vs. Allergy group)	*p* value
*Ccl11*	1.37	0.605
*Ccl24*	1.61	0.357
*Ccl26*	1.61	0.357

*Ccl11* CCL11/eotaxin-1; *Ccl24* CCL24/eotaxin-2; *Ccl26* CCL26/eotaxin-3

In experimental protocol 2, a comparison between the IL-23 and non-sensitized IL-23 groups showed a significantly increased fold expression of interleukin 1 receptor type 1 (*Il1r1*) in the IL-23 group (Table 3). By contrast, the non-sensitized IL-23 group exhibited significantly elevated expression of matrix metallopeptidase 3 (*Mmp3*), C-C motif chemokine ligand 7 (*Ccl7*), and C-C motif chemokine receptor 2 (*Ccr2*) (Table 4).

**Table 3:** Upregulated T-cell transcription factors in the interleukin (IL)-23 group compared to the non-sensitized IL-23 group

Symbol	Fold increase (IL-23 vs. Nonsensitized IL-23 group)	*p* value
*RorC*	3.98	0.011*
*GATA3*	1.94	0.367
*TBX21/T-bet*	1.33	0.757
Foxp3	1.33	0.757
*Il1R1*	1.91	0.014*

^*^ *p* < 0.05

*RorC* RAR-related orphan receptor gamma; *GATA3* GATA binding protein 3; *TBX21* T-box transcription factor 21; *T-bet* T-box expressed in T cells; *Foxp3* forkhead box protein P3; *Il1R1* IL-1 receptor type 1

**Table 4:** Downregulated inflammation-related factors in the interleukin (IL)-23 group compared to the non-sensitized IL-23 group

Symbol	Fold decrease (IL-23 vs. Non-sensitized IL-23 group)	*p* value
*MMP3*	10.96	0.033*
*CCL7/MCP-3*	7.43	0.034*
*CXCL5*	3.41	0.160
*CCR2*	3.28	0.046*
*CCL2/MCP-1*	3.08	0.156
*IL-1β*	2.52	0.157

^*^ *p* < 0.05

*MMP3* matrix metalloproteinase-3; *CCL7* C-C motif chemokine ligand 7; *MCP-3* monocyte chemotactic protein-3; *CXCL5* C-X-C motif chemokine ligand 5; *CCR2* CC chemokine receptor 2; *CCL2* C-C motif chemokine ligand 2; *MCP-1* monocyte chemotactic protein-1; *IL-1β* interleukin-1 beta

#### Quantitative PCR analysis

**Fig. 5:**
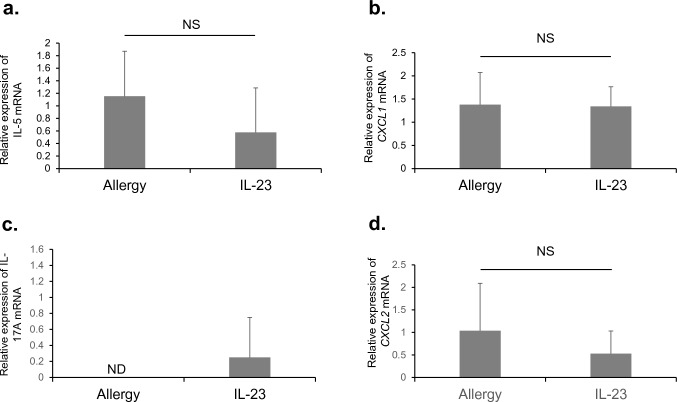
Cytokine mRNA expression in the conjunctival tissue of the allergy and interleukin (IL)-23 groups. mRNA expression levels of **a** IL-5, **b** chemokine (C-X-C motif) ligand (CXCL) 1, and **c** CXCL2, all mRNA expression levels do not differ between the allergy and IL-23 groups. **d** IL-17 mRNA expression is detectable in the allergy group; however, it is below the detection limit in the IL-23 group. *NS* not significant

Following experimental protocol 1, RT-PCR analysis showed IL-5 expression in both the allergy and IL-23 groups, with no significant difference between the groups (Fig. 5a). Similarly, CXCL1 and CXCL2 expression levels were not significantly different (Fig. 5b and c). However, IL-17A expression was detected only in the IL-23 group (Fig. 5d).

**Fig. 6:**
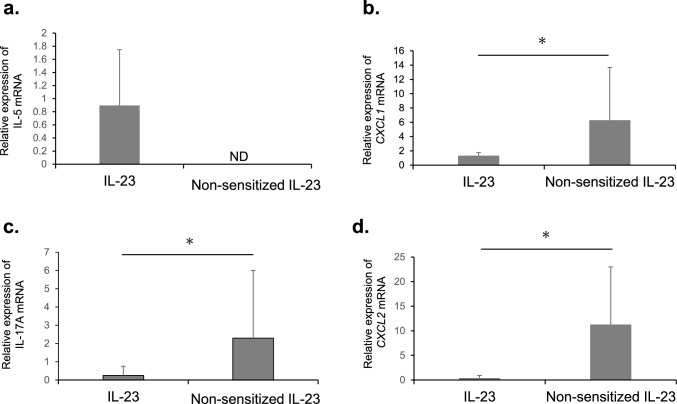
Cytokine mRNA expression in the conjunctival tissue of the allergy and interleukin (IL)-23 groups. **a** IL-5 mRNA expression is measurable in the IL-23 group; however it is below the detection limit in the non-sensitized IL-23 group. **b** Chemokine (C-X-C motif) ligand (CXCL) 1, **c** CXCL2, and **d** IL-17A mRNA levels are significantly higher in the allergy group than in the IL-23 group. ^*^p < 0.05; *NS* not significant; Mann–Whitney U test

In experimental protocol 2, IL-5 was detected only in the IL-23 group (Fig. 6a). CXCL1 (*p* < 0.05) and CXCL2 (*p* < 0.05) expression levels were significantly higher in the non-sensitized IL-23 group compared with the IL-23 group (Fig. 6b and c), as was IL-17A (*p* < 0.05) expression (Fig. 6d).

## Discussion

In this study, we investigated the effects of IL-23 on allergic conjunctival inflammation using a mouse model of OVA-sensitized EAC. IL-23 was injected into the eyelid, and the following findings were observed: (1) Neutrophil and eosinophil infiltration in the conjunctiva was higher in the IL-23 group (EAC with IL-23 injection) than in both the allergy group (EAC without IL-23) and the negative control group (non-treated) (Figs. 3 and 4). (2) PCR array analysis revealed a significant increase in the expression of CCL17/TARC in the IL-23 group compared with the allergy group (Table 1). (3) Th17-related genes, including *RorC* and *IL1R1*, were significantly higher in the IL-23 group compared with the non-sensitized IL-23 group (IL-23 without EAC) (Table 3). By contrast, *MMP-3*, *CCL7*, and *CCR2* levels were significantly higher in the non-sensitized IL-23 group (Table 4).

We compared the allergy and IL-23 groups to understand how IL-23 injection modulates allergic inflammation. Eosinophilic infiltration is a hallmark of Th2- and ILC2-mediated type 2 inflammation [17]. TARC, a chemokine that promotes Th2 migration via CC chemokine receptor 4 (CCR4) [18], was significantly upregulated in the IL-23 group. Serum TARC is also used as a biomarker for AD [19]. Therefore, the increased TARC expression in conjunctival tissue following IL-23 injection may have induced Th2 cell migration, exacerbating eosinophilic inflammation. Notably, *IL-5* mRNA levels were comparable between the IL-23 and allergy groups (Fig. 5a), suggesting that persistent type 2 inflammation is maintained despite IL-23 exposure. Although no significant differences were observed in *CXCL1/Groα/KC* and *CXCL2/MIP-2* mRNA—neutrophil-associated chemokines—between allergy and IL-23 groups (Fig. 5b and d), and IL-17A expression was detected only in the IL-23 group, but not in the allergy group (Fig. 5c). Therefore, IL-17A mRNA expression may be upregulated in the IL-23 group. IL-17A, a signature Th17 cytokine, is known to drive neutrophilic inflammation [20]. Therefore, increased neutrophil infiltration in the IL-23 group may reflect activation of the IL-23/Th17 immune axis. Given that mixed Th2 and Th17 responses are implicated in severe airway inflammation [7], the IL-23/Th17 axis may also contribute to AC disease severity, in addition to type 2 inflammation.

We also compared the IL-23 group with the non-sensitized IL-23 group to assess the effects of immunological background on IL-23-mediated responses. IL-23 primarily induces a Th17-differentiation environment, in which both Th1 and Th2 are suppressed [15]. In non-sensitized mice with healthy immune backgrounds, IL-23 induced upregulation of *MMP-3* and the *CCL7/CCR2* axis, along with *CXCL1*, *CXCL2*, and IL-17A expression (Table 4). MMP-3 is a known biomarker in rheumatoid arthritis [21], while *CCL2* and *CCL7/CCR2* expression regulate acute neutrophilic lung inflammation [22]. These confirm that IL-23 injection into the eyelids alone can trigger a neutrophil-driven inflammatory response. By contrast, the IL-23 group (with allergic inflammation) showed higher *IL1R1* and *RorC* expression than the non-sensitized IL-23 group. RorC is essential for Th17 differentiation in the presence of IL-6 and tumor growth factor-β [23], and IL-1β further enhances this process [24, 25]. Therefore, allergic inflammation may enhance the potential for Th17 differentiation. However, IL-17A levels were lower in the IL-23 group than in the non-sensitized IL-23 group, although IL-5, a major Th2 cytokine, significantly increased (Fig. 6a and c). This suggests that type 2 inflammation in the IL-23 group may have suppressed the Th17 response. Additionally, the *CCL7/CCR2* axis was less active in the IL-23 group compared with the non-sensitized IL-23 group. IL-23 stimulation can induce Th17 differentiation in CCR2-/- mice [26]; however, the mechanism underlying *CCL7/CCR2* downregulation in the context of type 2 inflammation remains unclear.

Increased local expression of IL-8 in the ocular surface and elevated circulating IL-17 levels are reported in pediatric patients with chronic allergic keratoconjunctivitis, including AKC and VKC [6]. These findings support the idea that mixed eosinophilic and neutrophilic inflammation may exacerbate allergic inflammation in chronic allergic keratoconjunctivitis. AD is considered a Th2-dominant disease, with Th2-induced allergic inflammation constituting the primary pathology. However, the immunological background of AD involves Th2-, Th1-, Th17-, and Th22-associated immune responses. Dupilumab, an anti-IL-4/IL-13 receptor antibody, is widely used to treat severe type 2-mediated allergic diseases. Dupilumab-associated conjunctivitis (DAC) is reported in patients with atopic dermatitis [27, 28]. Although several questions remain unanswered regarding DAC pathogenesis, our findings in mice may help elucidate its pathogenesis. When type 2 inflammation is alleviated by dupilumab, not only does the Th1/Th2 balance shift toward Th1, but Th17, which had been suppressed by Th2, may become activated and exacerbate neutrophil inflammation and conjunctivitis. Our findings offer a potential mechanistic explanation for DAC and underscore the relevance of the IL-23/Th17 axis in this context.

A major limitation of our study is that it focused on the acute inflammatory response to allergic inflammation caused by IL-23. Since severe ACD is associated with chronic inflammation, further studies using a chronic ACD model are needed to understand better the long-term contributions of IL-23 and the Th17 axis. Additionally, histological changes in the conjunctiva following IL-23 administration were observed as increased densities of eosinophils and neutrophils in conjunctival tissues. However, the molecular mechanisms underlying this increase in eosinophils and neutrophils could not be fully identified. Therefore, the increase in eosinophils and neutrophils warrants re-evaluation not only in terms of chemotaxis driven by eosinophil- and neutrophil-associated chemokines, but also regarding the possibility of local proliferation. Furthermore, since no inhibition assays using anti-IL-23 receptor antibodies or similar agents were performed, it remains unclear whether the increase in eosinophils and neutrophils in conjunctival tissue represents a direct or indirect effect of IL-23. Additional research on IL-23 inhibition should be pursued to translate the effects of IL-23 on the pathogenesis of allergic conjunctivitis, as revealed in this study, into clinical applications such as drug therapy.

IL-23 is a potential proinflammatory cytokine that promotes mixed eosinophilic and neutrophilic inflammation in severe allergic conjunctivitis; however, its Th17-driven effects appear to be suppressed by ongoing type 2 inflammation. These findings suggest a modulatory role of the IL-23/Th17 axis in influencing the severity and phenotype of allergic conjunctival inflammation.

## Data Availability

All data generated or analyzed during this study are included in this published article.
